# Mutational Analysis of Circulating Tumor Cells from Colorectal Cancer Patients and Correlation with Primary Tumor Tissue

**DOI:** 10.1371/journal.pone.0123902

**Published:** 2015-04-22

**Authors:** Anna Lyberopoulou, Gerasimos Aravantinos, Efstathios P. Efstathopoulos, Nikolaos Nikiteas, Penelope Bouziotis, Athina Isaakidou, Apostolos Papalois, Evangelos Marinos, Maria Gazouli

**Affiliations:** 1 Department of Basic Medical Sciences, Laboratory of Biology, School of Medicine, University of Athens, Athens, Greece; 2 Second Department of Medical Oncology, Agioi Anargiroi Cancer Hospital, Kifisia, Athens, Greece; 3 Second Department of Radiology, School of Medicine, University of Athens, Athens, Greece; 4 Second Department of Propedeutic Surgery, University of Athens School of Medicine, Laiko General Hospital, Athens, Greece; 5 Institute of Nuclear and Radiological Sciences and Technology, Energy and Safety, N.C.S.R. 'Demokritos', Athens, Greece; 6 Exprerimental Research Center ELPEN Pharmaceuticals, Pikermi, Attiki, Greece; University of Navarra, SPAIN

## Abstract

Circulating tumor cells (CTCs) provide a non-invasive accessible source of tumor material from patients with cancer. The cellular heterogeneity within CTC populations is of great clinical importance regarding the increasing number of adjuvant treatment options for patients with metastatic carcinomas, in order to eliminate residual disease. Moreover, the molecular profiling of these rare cells might lead to insight on disease progression and therapeutic strategies than simple CTCs counting. In the present study we investigated the feasibility to detect KRAS, BRAF, CD133 and Plastin3 (PLS3) mutations in an enriched CTCs cell suspension from patients with colorectal cancer, with the hypothesis that these genes` mutations are of great importance regarding the generation of CTCs subpopulations. Subsequently, we compared CTCs mutational status with that of the corresponding primary tumor, in order to access the possibility of tumor cells characterization without biopsy. CTCs were detected and isolated from blood drawn from 52 colorectal cancer (CRC) patients using a quantum-dot-labelled magnetic immunoassay method. Mutations were detected by PCR-RFLP or allele-specific PCR and confirmed by direct sequencing. In 52 patients, discordance between primary tumor and CTCs was 5.77% for KRAS, 3.85% for BRAF, 11.54% for CD133 rs3130, 7.69% for CD133 rs2286455 and 11.54% for PLS3 rs6643869 mutations. Our results support that DNA mutational analysis of CTCs may enable non-invasive, specific biomarker diagnostics and expand the scope of personalized medicine for cancer patients.

## Introduction

Colorectal cancer (CRC) remains a leading cause of mortality worldwide [1]. During the natural course of the disease, approximately 15% to 25% of patients will present metastases mainly to the liver at diagnosis and another 25% to 50% will develop metachronous metastasis following resection of the primary tumor [2]. Undoubtedly, metastatic disease is the most common cause of cancer-related death in patients with solid tumors like colorectal cancer. Metastasis is associated with the presence of circulating tumor cells (CTCs) in the peripheral blood of cancer patients [3]. Additionally, the presence of CTCs before and after the adjuvant chemotherapy is associated with poor clinical outcome [4]. The term CTC includes all cell subpopulations which are considered as foreign entities in the blood having cancerous characteristics such as cancer stem cells, tumor amplifying cells and tumor initiating cells arise from epithelial cancer cells of the primary tumor undergoing epithelial mesenchymal transition (EMT) program [5–7].

Nowadays the use of new antitumoral drugs for mCRC such as the epidermal growth factor receptor-targeted monoclonal antibodies (EGFR-mAbs) and tyrosine kinase inhibitors have significantly improved the treatment of colorectal disease patients [8, 9]. Concerning the EGFR-mAbs therapy, only a small proportion (10–20%) of mCRC patients respond, which is in part due to activating mutations in genes downstream of the EGFR-receptor [10].

Currently, KRAS mutational status is the only biomarker predictive of the response to therapy using EGFR-mAbs that have been validated for clinical practice in mCRC [11, 12]. However, not all mCRC patients with wild-type KRAS respond to EGFR-mAb treatment, which may be due to alterations in other genes like BRAF, PIK3CA, etc [13]. It is interesting to notice that several studies reported discordance in the KRAS mutational status between the primary tumor and the metastatic tissues [14]. In addition to KRAS, the BRAF V600E mutation is currently also used as a predictive mutation regarding the response to EGFR-mAb therapy [15]. Today, KRAS and BRAF mutational status is determined in the primary tumor tissues before treatment initiation, however several problems arise. Usually, primary tumor tissue is of insufficient quality, tissue has been obtained longtime ago before the diagnosis of metastatic disease, biopsies from metastatic sites are not always feasible and most important mutation status of the primary and metastatic lesions can be changed during the course of disease and therapy [16, 17]. To overcome these problems, several studies suggest that the characterization of the mutation status characterization of CTCs that can be repeatedly performed in a way that could serve as a marker of micrometastatic tumor load associated with patients' prognosis and accurately predict the effectiveness of therapy in several cancers [18–20]. Recently numerous studies determined KRAS and BRAF mutations in the CTCs of patients with mCRC, suggesting that CTCs may represent an alternative non-invasive procedure and their analysis may be representative of the current disease status of the patient [16, 17, 21]. However, the mutational status in genes related to different CTCs subpopulations, such as cancer stem cells and cells with EMT phenotype, are excluded in these studies. At the same time antibodies against epithelial adhesion molecule (EpCAM) and cytokeratins are mainly used to capture and detect CTCs, but during epithelial to mesenchymal transition, the expression of such epithelial markers on CTCs, may be downregulated and become undetectable [22, 23]. Most importantly, the aforementioned studies are mainly focused in predicting response to EGFR-mAb treatment.

Bevacizumab, a monoclonal human antibody targeting Vascular Endothelial Growth Factor (VEGF), has also proven its efficacy in CRC and is given as first line chemotherapy in patients with mCRC [24]. CD133, a pentaspan transmembrane protein, has been used by several groups to identify cancer stem cells in colorectal cancer [25, 26] and Pohl *et al*. [27] suggested that CD133 might be a predictive marker for standard first-line bevacizumab-based treatment in mCRC.

Additionally, recent studies have indicated the presence of a subpopulaton in CTCs that show features of EMT in patients with epithelial origin tumors [28, 29] and the importance of developing new markers to capture and detect CTCs, markers which are not downregulated by the induction of EMT. Very recently Yokobori *et al*. [30] suggested plastin3 (PLS3) as a new marker for mCRC, CTCs. Furthermore, Ning *et al*. [29] suggested that PLS3 rs6643869 SNP could serve as stage-specific molecular predictor of tumor recurrence in stages II/III CRC patients as well as a potential therapeutic target.

Thus CTCs detection and mutational analysis has enormous potential for metastases prediction, monitoring response of patients to therapy and early prediction of relapse, however the current markers used for the enrichment and their detection often miss the most aggressive subpopulations of cancer stem cells and EMT-induced CTCs [31]. In the present study we examined the feasibility to detect KRAS, BRAF, CD133 and PLS3 mutation in a CTC cell suspension which was enriched using markers of each subpopulation, from CRC patient’s peripheral blood. Thus, we detected and isolated a pure CTC population that contains CTCs of epithelial origin, stem cell-like cells and cells showing features of EMT. Additionally, a comparison between the mutational status of CTCs and the corresponding tumor tissue was performed.

## Material and Methods

### Patients

Peripheral blood samples and tumor tissue biopsies were obtained from 52 CRC patients before initiation of any treatment, who gave informed consent to be included in this study (S1 Table). Written informed consent was obtained from each patient or their families concerning the samples involved in the study. The research was approved by the Ethics committee of Atticon University Hospital, School of Medicine, University of Athens, Greece under the general title “Molecular Characterization of Circulating Tumor Cells” (1586/27/1/14). Peripheral venous blood was sampled immediately after patients were anaesthetized and prior to the commencement of surgery. In all patients, an intravenous cannula was used to collect blood into 7-mL vacutainers containing sodium ethylenediaminetetraacetic acid (EDTA), discarding the first 7-mL aliquot of blood to reduce the risk of contamination of blood by skin epithelial cells. Three 30-mL samples were then collected at one-minute intervals. Human peripheral blood monocellular cells (PBMCs) were isolated from peripheral blood using Ficoll-Hypaque PLUS reagents (Amersham Bioscienses, Little Chalfont, NA, UK). Cells were counted manually using a Burker-Turk haemocytometer. Trypan blue (0.4%, Sigma, LΟ, UK) exclusion test was used to ensure cell viability was above 90% in experiments [32]. All patients had detectable number of CTCs as verified by FACS (>2 cancer cells). Primary tumor tissue samples were obtained during surgery, frozen immediately in liquid nitrogen and stored at -80°C until DNA extraction.

### Enrichment of CTCs

The enrichment of tumor cells from whole blood was performed by density gradient centrifugation using a *ficoll*–hypaque solution (Histopaque-1077, Sigma-Aldrich Chemie GmbH) and by CD45 depletion of the leukocyte fraction using the Human Dynabeads® CD45 kit (Life Technologies GmbH, Darmstadt, DE) following the manufacturers' instructions. The remaining cell fraction (CD45- fraction) that contains the CTC population was used for further analyses and enrichment using a quantum-dot-labelled magnetic immunoassay method developed in our laboratory [32]. The sensitivity and specificity of this method is already standardized. The double-step enrichment of this method offers the possibility to isolate a pure population of CTCs with no hematopoietic contamination that could alter our data, as validated by FACS as previously described [32]. Specifically, in the present study streptavidin-coated magnetic beads (MBs) (Life Technologies) functionalized with the biotinylated mouse anti-human EpCAM (GTX72682, Acris Antibodies Inc. Acris GmbH, San Diego, CA, USA), mouse anti-human Vimentin (BM5501B Acris Antibodies Inc.) were used in order to collect in a cell suspension all the CTCs including CSCs, cells showing features of EMT and cancer cells of epithelial origin [19, 33]. It is well known that immunomagnetic EpCAM based methods are used to enrich CTCs including cancer stem cell populations (CSCs) in several cancers [34, 35] and recent studies showed that a subpopulation of CTCs with EMT phenotype expresses vimentin on the cell surface [36]. Further enrichment of the CTCs suspension and subsequent imaging was performed, by targeting these complexes with functionalized Quantum dots (Qdots, Life Technologies): Qdot655-anti-CK19 (SM182B, Acris), Qdot525-CD133 (AC133, Biocompare, South San Francisco, CA, USA) and Qdot605-CD29 (SM1578B, Acris) that specifically recognize CTCs, leading to the detection of 3 different fluorescent signals under a fluorescence microscope [32, 37, 38]. Samples that did not contain cells of epithelial origin (CK19+), stem-cell like cells (CD133+) and cells showing features of EMT (CD29+) (all sub-populations of interest), were not analyzed and not included in our study.

### DNA isolation from enriched CTC samples and tissues

DNA from the enriched CTC samples and from the corresponding tissue samples was isolated using the NucleoSpin Tissue kit (Macherey-Nigel, GmbH & Co. KG, Düren, Germany) according to the manufacturers' instructions.

### Genotyping

All DNA samples were analyzed for mutations in KRAS (codons 12 and 13), BRAF (V600E), CD133 (rs3130 and rs2286455) and PLS3 (rs6643869). KRAS and BRAF mutations were determined as previously described [39, 40]. Briefly, KRAS mutations were analyzed by PCR restriction fragment length polymorphism (RFLP) assay, using the primers: KRAS12F: 5’ ACTGAATATAAACTTGTGGTAGTTGGACCT 3’; KRAS12R: 5’ CTGTATCAAAGAATGGTCCTGCACCAGTA 3’; KRAS13F: 5’ GTACTGGTGGAGTATTTGATAGTGTATTAA 3’; KARS13R: 5’ GTATCGTCAAGGCACTCTTGCCTAGG 3’. Regarding the detection of codon 12 mutation, the KRAS12F primer used generates a recognition site for *Mva*I restriction enzyme. *Mva*I digestion of wild-type codon 12 allele yields two bands of 133 and 29 bp, while the mutant type remains intact (162 bp). The KARS13R primer used for the detection of codon 13 mutation, generates a *Hae*III recognition site. *Hae*III digestion of wild-type codon 13 allele yields fragments of 85, 48 and 26 bp while the mutant results in only two fragments of 85 and 74 bp. The V600E mutation results in an amino acid substitution at position 600 in BRAF, from a valine (V) to a glutamic acid (E). The genotyping was performed by allele-specific PCR using the primers: BRAF-A: 5’ TCATAATGCTTGCTCTGATAGGA 3’; BRAF-T: 5’ TCATAATGCTTGCTCTGATAGGT 3’; BRAF-R: 5’ GGCCAAAAATTTAATCAGTGGA 3’. CD133 (rs3130 and rs2286455) genotyping was performed as described by Pohl *et al*. [27]. CD133 (rs3130 and rs2286455) genotyping was performed using the following primers: 3130F: 5’ AGAACTGCAATCTGCACATGA 3’; 3130R: 5’ TGATCAGCAATGAAGAACTGG 3’; 2286455F: 5’ ACGCCTCTTTGGTCTCCTTG 3’; 2286455R: 5’ TCCATCCCAAGTCCCTTTAG 3’. Regarding the rs3130, an *EcoR*I recognition site was generated, yielding two fragments of 50 bp for mutant allele and a 100 bp fragment for the wild-type allele, while an *Mbo*I recognition site was generated for rs2286455. Regarding the PLS3 (rs6643869) mutation genotyping was performed by allele-specific PCR using the primers: PLSG: 5’ TTTAGATATATCCAAGGCCG 3’, PLGA: 5’ TTAGATATATCCAAGGCCA 3’ and PLSR: 5’ CCCTACTACTATTTCCATGACA 3’. All reactions were performed in a 50 μl total reaction volume, with 300–500ng DNA template 0.2mM of each dNTP, 20mM Tris HCl (pH 8.4), 50mM KCl, 1.5mM MgCl, 1.5 units of Taq polymerase and 10 pmol/μl of each primer. PCR conditions were programmed at 95°C for 5min followed by 35 amplification cycles: for KRAS codon 12 and 13, denaturation at 95°C for 30 s, annealing at 55°C for 30 s, extension at 72°C for 30 s. For rs3130 and rs2286455 the annealing was changed to 60°C, while for PLS it was changed to 55°C. For BRAF, annealing was programmed at 66°C for 1 min. In all cases the results were confirmed by direct sequencing.

## Results

**Fig 1:**
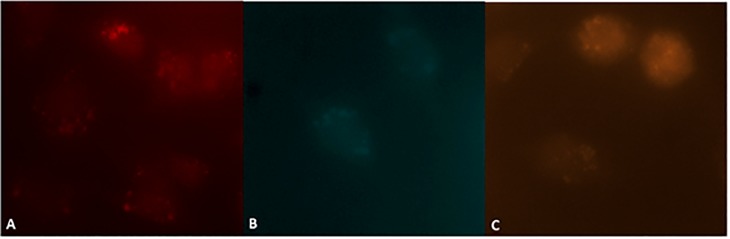
QD-labeled CTC subpopulations. Representative fluorescent images of QD-labeled CTC subpopulations. A. Qdot655-anti-CK19 labelled epithelial origin CTCs, B. Qdot525-CD133 labelled stem-cell CTCs, and C. Qdot605-CD29 labelled EMTs. All images were acquired with an oil immersion 40× objective.

Fifty-two colorectal cancer patients with detectable number of CTCs (>2 cells), as verified by FACS, were included in the study. The CTCs samples contained epithelial origin, stem-like cell and possible EMTs as demonstrated with QD-labelling (Fig 1).

Detailed patient characteristics are depicted in Table 1.

**Table 1:** Histopathological characteristics of colorectal cancer patients.

****Characteristics****	****No. of patients (n = 52)****
Age (median, range)	63 (35–82)
**Sex**	
Female	19
Male	33
**Primary tumor location**	
Rectum	11
Right colon	12
Left colon	29
**Differentiation**	
Well	6
Moderate	40
Poor	6
**TNM stage**	
I	0
II	6
III	24
IV	22

The comparison of mutation status between matched primary tumors and CTCs mixed suspension samples is presented in Table 2. Twenty-one patients were found to be homozygotes for KRAS codon 12 mutations, in CTCs and tissue, whereas 8 were found heterozygotes in CTCs and 5 in tissue samples. None of the patients were found to carry mutation in KRAS codon 13. More specifically, 5.77% discordance between the primary tumor KRAS status and the CTC KRAS status was found in the samples tested. Specifically, 3 patients who demonstrated a KRAS wild-type in the primary tumor were found to have a mutated KRAS codon 12 allele in the CTC enriched cells fraction.

**Table 2:** Comparison of mutation status between matched primary tumor and CTC subpopulations.

Patient	Primary tumor						CTCs					
	KRAS-12	KRAS-13	BRAF V600E	CD133rs3130	CD133 rs2286455	PLS3 rs6643869	KRAS-12	KRAS-13	BRAF V600E	CD133rs3130	CD133 rs2286455	PLS3 rs6643869
1	WT^a^	WT	WT	CC	CC	GG	G12V	WT	WT	CC	CC	GG
2	WT	WT	WT	CC	CC	AA	WT	WT	WT	CC	CC	AA
3	V12V	WT	WT	CT	CC	GA	V12V	WT	WT	CT	CC	GA
4	WT	WT	V600E	CC	CC	GG	WT	WT	V600E	CC	CC	GG
5	V12V	WT	WT	CC	CT	AA	V12V	WT	WT	CT	CT	AA
6	G12D	WT	WT	CC	CC	GA	G12D	WT	WT	CC	CT	GA
7	WT	WT	WT	CC	CC	GG	WT	WT	WT	CC	CC	GG
8	WT	WT	WT	CT	CC	AA	WT	WT	WT	CT	CC	AA
9	WT	WT	WT	CC	CC	GG	G12V	WT	WT	CC	CC	GG
10	G12V	WT	WT	CT	CT	GA	G12V	WT	WT	CT	CT	GA
11	WT	WT	WT	CC	CC	AA	WT	WT	WT	CC	CC	AA
12	V12V	WT	WT	CC	CT	GG	V12V	WT	WT	CC	CT	GG
13	WT	WT	V600E	CC	CC	AA	WT	WT	V600E	CC	CC	AA
14	WT	WT	WT	CC	CC	GA	WT	WT	WT	CT	CC	GA
15	D12D	WT	WT	CT	CC	AA	D12D	WT	WT	CT	CC	AA
16	V12V	WT	WT	CC	CT	GG	V12V	WT	WT	CC	CT	GG
17	V12V	WT	WT	CT	CC	AA	V12V	WT	WT	CT	CC	AA
18	WT	WT	V600E	CC	CC	GA	WT	WT	WT	CC	CC	GA
19	WT	WT	WT	CC	CC	GG	WT	WT	WT	CT	CC	GG
20	G12V	WT	WT	CT	CC	AA	G12V	WT	WT	CT	CC	AA
21	WT	WT	WT	CC	CC	GG	WT	WT	WT	CC	CT	GA
22	V12V	WT	WT	CC	CC	GG	V12V	WT	WT	CC	CC	GG
23	D12D	WT	WT	CC	CC	GG	D12D	WT	WT	CC	CC	GG
24	WT	WT	WT	CC	CC	GA	WT	WT	WT	CT	CC	GA
25	WT	WT	WT	CC	CT	GG	WT	WT	WT	CC	CT	GG
26	D12D	WT	WT	CC	CC	GG	D12D	WT	WT	CC	CC	GG
27	C12C	WT	WT	CT	CT	AA	C12C	WT	WT	CT	CT	AA
28	WT	WT	WT	CT	CC	GG	WT	WT	WT	CT	CC	GG
29	G12V	WT	WT	CC	CC	GG	G12V	WT	WT	CC	CC	AA
30	D12V	WT	WT	CT	CT	GA	D12V	WT	WT	CT	CT	GA
31	V12V	WT	WT	CC	CC	AA	V12V	WT	WT	CC	CC	AA
32	WT	WT	WT	CC	CC	GA	G12D	WT	WT	CC	CC	GG
33	WT	WT	V600E	CT	CC	GA	WT	WT	V600E	CT	CC	GA
34	WT	WT	WT	CC	CC	AA	WT	WT	WT	CT	CC	GG
35	V12V	WT	WT	CC	CC	GG	V12V	WT	WT	CC	CC	GG
36	V12V	WT	WT	CT	CC	GG	V12V	WT	WT	CT	CT	GG
37	V12V	WT	WT	CC	CC	GG	V12V	WT	WT	CC	CC	GG
38	WT	WT	WT	CC	CC	GG	WT	WT	WT	CC	CC	GG
39	WT	WT	WT	CT	CC	AA	WT	WT	WT	CT	CC	AA
40	V12V	WT	WT	CC	CC	GG	V12V	WT	WT	CC	CC	AA
41	V12V	WT	WT	CC	CC	GG	V12V	WT	WT	CC	CC	GG
42	WT	WT	V600E	CC	CC	GG	WT	WT	V600E	CC	CC	GG
43	V12V	WT	WT	CC	CC	GG	V12V	WT	WT	CT	CC	GG
44	D12D	WT	WT	CC	CC	GG	D12D	WT	WT	CC	CC	GG
45	WT	WT	WT	CC	CT	GG	WT	WT	WT	CC	CT	GG
46	WT	WT	WT	CC	CC	GG	WT	WT	WT	CC	CC	GG
47	D12D	WT	WT	CT	CC	GG	D12D	WT	WT	CT	CC	GG
48	WT	WT	WT	CC	CC	GG	WT	WT	WT	CC	CC	GG
49	V12V	WT	WT	CC	CC	GG	V12V	WT	WT	CC	CT	GG
50	WT	WT	V600E	CT	CC	GG	WT	WT	WT	CT	CC	AA
51	WT	WT	WT	CC	CC	GG	WT	WT	WT	CC	CC	GG
52	D12D	WT	WT	CC	CC	GG	D12D	WT	WT	CC	CC	GG

^a^WT: wild-type

The V600E BRAF mutation was present in 6 tissue samples of CRC patients, and in 4 CTCs samples respectively. Thus, the discordance between the primary tumor and the CTC BRAF status was 3.85%. Two patients with a BRAF mutation in the primary tumor did not carry a mutant allele in their CTC subpopulation. All carriers were heterozygotes for BRAF mutation and they did not carry a KRAS mutation. Concerning the CD133 mutations, the rs3130 C/T genotype was found in 14 tissue samples and in 20 CTCs samples, with an 11.54% discordance between the primary tumor and CTCs mutational status. The rs3130 mutated allele (T) found in 6 CTCs samples, corresponded to wild-type primary tumors. In addition, the rs2286455 T mutated allele was found in 12 CTCs samples, while in 4 cases mutation was detected only in CTCs (discordance 7.69%). Finally, a discordance of 11.54% for PLS3 rs6643869 A/G mutation was observed. Twenty-three patients were carriers of the A mutated allele (14 had AA and 9 GA genotypes). Four patients had mutations only in CTC populations whereas in 2 the mutation was found only in primary tumor samples.

## Discussion

It is well accepted that CTCs in the bloodstream play a crucial role in establishing metastases. Recently, the clinical value of CTCs as a biomarker for early cancer detection, diagnosis, prognosis, prediction, stratification and therapeutic guidance has been widely explored [41–43]. Tumor biopsy is indisputably an invasive approach, not always practicable. It captures only a small tissue sample at a single time point, which is not representative of all tumor cell populations existing in a tumor and has little utility for monitoring changes of tumor properties over time or in response to treatment. Additionally, the currently used standard methods for preservation of tissue samples, such as formalin-fixed, paraffin-embedded samples, are not suitable for later analysis, due to DNA loss and fragmentation [44]. For these reasons, molecular characterization of CTCs in blood offers an alternative or supplementary non-invasive method to tissue biopsy.

To date the majority of studies are focused on determining KRAS and BRAF mutations in CTCs, in order to predict the lack of therapeutic response to anti-EGFR monoclonal antibodies [16, 17, 21]. Nevertheless, most of these studies are based on the use of epithelial markers for CTCs isolation, when at the same time it is well known that during epithelial to mesenchymal transition, the expression of epithelial markers on CTCs, such as epithelial cell adhesion molecule (EpCAM) and cytokeratin (CK) may be down-regulated and become undetectable [37]. Therefore, accurate detection of CTCs based on morphological and immunophenotypical profiling is still challenged [45]. Recent data show that apart from KRAS and BRAF mutations, mutations in genes related to colorectal cancer stem cells or cells that undergo EMT, such as CD133 and PLS3, might have therapy-predictive value and clinical significance [27, 29].

In the present study, we were able to detect KRAS, BRAF, CD133 and PLS3 mutations in cell suspension enriched for all CTCs populations and in the corresponding primary tumor of 52 patients with colorectal cancer. The concordance between primary tumor and CTCs was high (88.46%- 96.15%) and concerning KRAS and BRAF mutations comparable to previous studies [16, 21]. The discordance of mutations between primary tumors and CTCs might be explained by the fact that in a 5–10% of cases, the mutational status is heterogeneous and may vary between the primary tumor and the metastatic cells [46]. However, it is not known whether a wild-type primary tumor will lead to mutated CTCs and metastases. For example, the discordance of KRAS and BRAF mutations due to genetic diversification of metastatic cells compared to their primary tumor, may explain the lack of efficacy and the emergence of subsequent resistance when treating metastatic disease with EGFR monoclonal antibodies.

To our knowledge, this is the first study that describes a relationship with germline variations in CD133 and PLS3 in relation to CTCs and primary tumor. CD133 is a widely used surface protein for the isolation of colorectal, lung, breast, ovarian, oral, and pancreatic cancer stem cell-like cells [47–51]. Regarding colorectal CD133+ cancer stem cell-like cells, they are correlated with the invasiveness and differentiation of colorectal tumors, while Pohl *et al*. correlated their pharmacogenetic profiling with RR (response rate) and PFS (progression-free survival) in patients with colorectal cancer treated with bevacizumab. On the other hand, PLS3 is a novel marker for CTCs in CRC [30]. The aberrant expression of PLS3 was associated with copy number gain in CTCs from primary tumors and was involved in the regulation of the EMT, contributing to a poor prognosis in CRC patients [30, 52]. Thus, PLS3 needs to be further investigated as it seems to be a useful prognostic biomarker.

Furthermore, the comparison of PLS3 and CD133 mutations in CTCs and the corresponding primary tumor might elucidate the complex mechanism of the metastatic process. According to the “CSCs hypothesis” heterogeneity within the population of tumor cells develops during tumor progression due to the effect of genomic instability and the accumulation of mutations, while during the clonal selection tumor cells gain which can disseminate and form metastases. It is further implied a preexistence of functional heterogeneity within tumor cells with a discrete subpopulation of CSCs, able to initiate and maintain tumor growth. CSCs do not necessarily arise from normal tissue stem cells. They can also originate from more differentiated progenitor cells which underwent transformation [52]. Alternatively, CSCs may arise through an EMT process from transformed epithelial cells and achieve migratory and tumor-spreading properties. Thus, CTCs and CSCs are not necessarily separate populations of cancer cells, as CTCs generated in the process of EMT can bear features characteristic of CSCs [52]. In this study we demonstrated that CTCs and corresponding primary tumors have discordance in the mutation status (11.54% for rs3130 and PLS3), suggesting that the genotype conversion of these genes may play a significant role in the generation of CSCs via the EMT mechanism during the metastatic procedure. Furthermore, we noticed that the discordance in rs 3130 mutational status was due to patients with poor differentiation in stage IV and thus confirming the role of CD133+ cells regarding the invasiveness and differentiation of colorectal tumors.

Our study was initiated to investigate the feasibility to determine CTCs mutational status and to examine if CTCs molecular characterization could represent an alternative to invasive procedures in the future. A QD magnetic immunoassay method was used in this study in order to further enrich the CTC suspension and to verify that this subpopulation-enriched CTC suspension includes all three CTCs subpopulations of interest. This method is proven to be sensitive and specific and we think that its use will facilitate any possible future incorporation at point-of-care procedures [32]. The use of QDs in this approach bypasses the disadvantages of fluorescent dyes often incorporated into immuno-detection tests such as rapid photobleaching, narrow excitation spectrum and low signal intensity. Furthermore, due to the high extinction coefficient and high quantum yield, QDs can provide the realistic quantitative estimates of the non-predominant targeted proteins at high sensitivity [32]. This method can be easily extended to the detection of any other tumor as the adaptation of the method would require only the incorporation of specific antibodies for the cancer or disease in question and allows the intact isolation of cells for further analysis (e.g. cell count, characterization, DNA analysis, microscopic techniques etc). Our ultimate objective is to isolate and further functionally characterize those CTCs by examining their chemotherapeutic efficacy and metastasis potential. The detection and characterization of CTCs that show an EMT or stem cell-like metabolism could be a powerful diagnostic tool for patient stratification, the early determination of therapy failure, or the potential risk of resistance to a given therapeutic intervention. Conversely, new therapeutic strategies consisting of molecular targeting of signal transduction pathways activated in cancer stem cells have to be developed to eliminate minimal residual disease.

## Supporting Information

S1 Table Detailed patients characteristics. (XLS)
